# Supplementary material for: RefPlantNLR is a comprehensive collection of experimentally validated plant disease resistance proteins from the NLR family
Source: PLoS Biol. 2021 Oct 20;19(10):e3001124. doi: 10.1371/journal.pbio.3001124 (PMC8559963; doi:10.1371/journal.pbio.3001124)
Supplement: S19 Dataset — This file contains Fig 4 in PDF format. (PDF) [file pbio.3001124.s031.pdf]

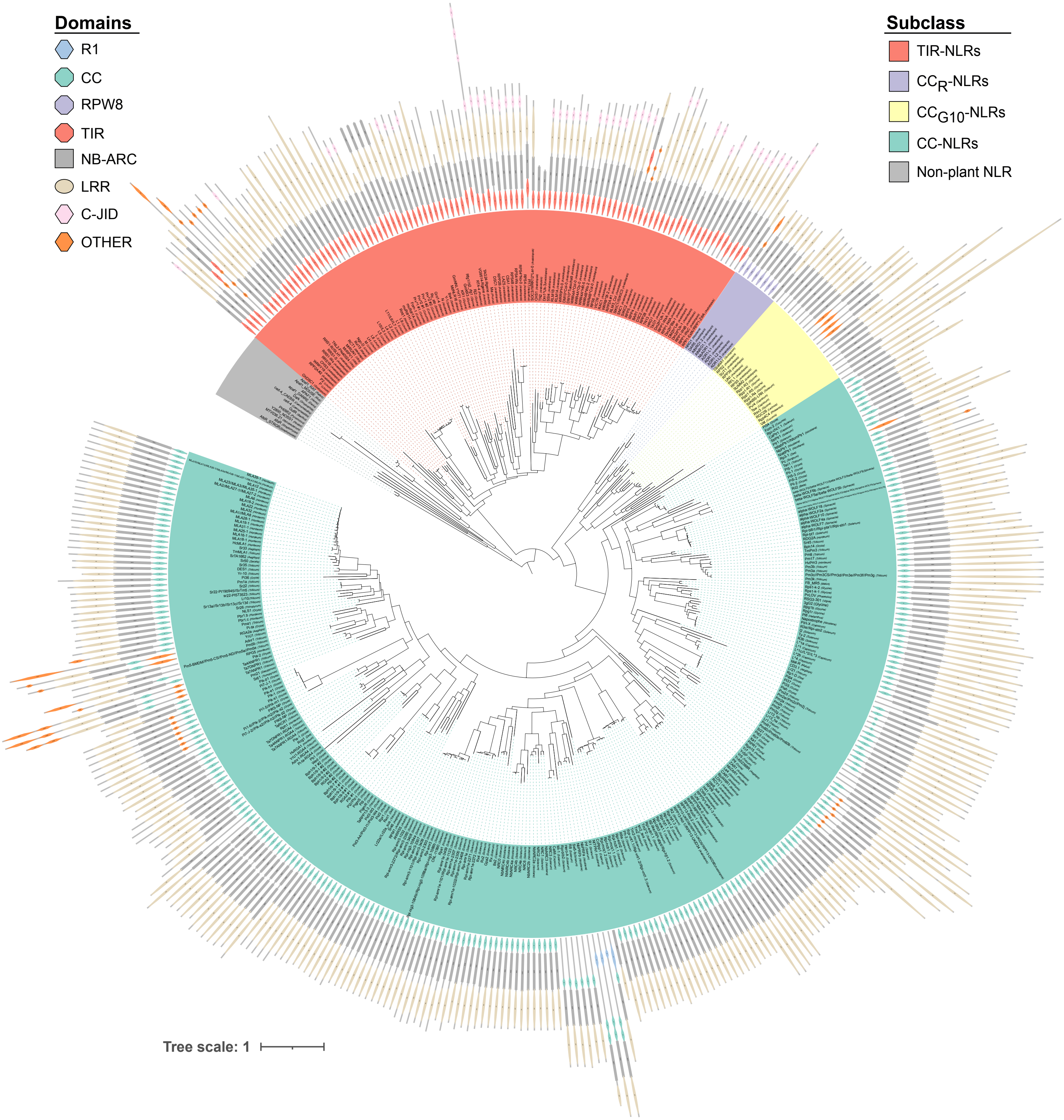

Domains
R1
CC
RPW8
TIR
NB-ARC
LRR
C-JID
OTHER
Subclass
TIR-NLRs
CCR-NLRs
CCG10-NLRs
CC-NLRs
Non-plant NLR
Tree scale: 1
