# Supplementary material for: RefPlantNLR is a comprehensive collection of experimentally validated plant disease resistance proteins from the NLR family
Source: PLoS Biol. 2021 Oct 20;19(10):e3001124. doi: 10.1371/journal.pbio.3001124 (PMC8559963; doi:10.1371/journal.pbio.3001124)
Supplement: Attachment — Submitted filename: 20210729_RefPlantNLR_Reviews.pdf [file pbio.3001124.s037.pdf]

Dear Roli and Ines,

Thank you very much for your evaluation of our manuscript "RefPlantNLR: a comprehensive collection of experimentally validated plant NLRs" for consideration as a Methods and Resources at PLOS Biology.

We appreciate the overall positive feedback of the four independent reviewers. We have endeavoured in this revision to address as much as possible the pertinent points they raised. Please see the point-by-point 'Response to Reviewers' attached to this file. Specifically, we have made an R package called refplantnlR (https://github.com/JKourelis/refplantnlR) which provides with an interactive manner to access the data from the paper through the R interface, something most biologists should be familiar with. This R package comes pre-packaged with the RefPlantNLR dataset, as well as the NLRs extracted from the plant NCBI RefSeq proteomes using NLRtracker.

Best regards,

Sophien Kamoun

****
REVIEWERS' COMMENTS:

Reviewer #1:
[identifies himself as Michael Seidl]

The research article 'RefPlantNLR: a comprehensive collection of experimentally validated plant NLRs' by Kourelis and colleagues report on a collection of experimentally validated plant NLR immune receptors. The authors exploit this resource to i) describe features of functionally characterized NLR immune receptors, and ii) to benchmark tools used to predict NLRs from plant genomes. Based on these results, the authors propose a novel NLR prediction tool, which is based on established bioinformatic tools and resources. Tracing the evolution and diversity of NLRs in plants is a prerequisite to better understand the immune system in plant model systems and to develop disease resistant crops. Thus, the here presented dataset and the novel NLR prediction tools will be very interesting resources for the plant community with high potential impact on supporting and guiding future research.

We thank the reviewer for the nice summary of this work.

The paper is well written with clear and informative figures; see few comments for further improvement below. Accessibility of the underlying data, description of the performed analyses, and the script for NLRtracker is exemplary as all of these are available as supplementary data, as are the relevant sequences in flat files. This should make the data accessible and useful for a wide range of plant biologists. The authors could consider making these data also accessible via a publicly available online database, which could also serve as a dynamic community hub to i) submit functionally

characterized NLRs, ii) retrieve complete RefPlantNLRs sets or iii) species/lineage specific subsets. Furthermore, this database could even house complete predicted NLR repertoires from species with complete genomes. This is of course out of the scope of the current manuscript but would be an incredibly useful resource for the community.

We thank the reviewer for these suggestions. Regarding making the data accessible through a publicly available database: we have currently uploaded all the flat files to Zenodo. This ensures that the different versions can be easily distributed, get a DOI which enhances reproducible research, and can be easily updated. Additionally, this should ensure that the data is available indefinitely as Zenodo is maintained by CERN. Please also note that this project prompted us to create an OpenPlantNLR community, which by now not only groups resources associated with this study but also various datasets from other groups. We hope that these efforts would further prompt the NLR community to open share datasets and resources.

To make the domain architecture annotation more easily accessible we made an R package called refplantnlR (https://github.com/JKourelis/refplantnlR). This package can either plot the RefPlantNLR or RefSeq NLR domain architecture with which it comes pre-packaged, or when given the output of NLRtracker the NLRtracker domain architecture.

Regarding predicted NLR repertoires: running NLRtracker requires a Linux environment due to the InterPro dependency. This might make this tool not directly accessible to those not familiar with Linux. In order to overcome this obstacle, we have now run the latest version of NLRtracker on all plant NCBI RefSeq proteomes and provide the extracted NLRs and their annotation and domain architecture as well as the extracted, NB-ARC domains as an additional supplementary information (119 species, of which 7 species with two different cultivars (total 126 genomes), 78963 NLRs belonging to 46210 distinct loci) (Supplemental dataset S17, **NEW**, Supplemental table S5, **NEW**). In our future Zenodo updates of this project we will aim to add more species as these are sequenced and/or other popular annotations. We chose the NCBI RefSeq annotation as these are generated with (different versions of) the same pipeline which makes it easier to compare between species. This should provide an important additional resource next to the RefPlantNLR dataset. We have done a basic analysis of the number of NLR loci/species (Figure S6, **NEW**), and the percentage containing potential integrated domains (Figure S6, **NEW**, Supplemental dataset 18, **NEW**). Additionally, NLRtracker annotates integrated domains, and for 50% of them it assigns them to a specific subclass (e.g. HMA, Protein kinase, transposon) while the remainder is classified as other, which we represent in Figure S7, **NEW**.

As discussed by the authors, the description of the generic NRL features is clearly biased towards the subset of species for which functionally characterized immune receptors are available (see also comment below). Thus, the most interesting aspect of the here presented work is the application of the RefPlantNLR set to benchmark NLR predictors, which is relevant as many predictors have been only benchmarked with very few or selected species (e.g., Arabidopsis). Based on the benchmark results and especially due to the inability of most tools to reliably classify the domain architecture of NLRs (Table 1; Figure 5B), the authors propose a novel tool - NLRtracker. The authors benchmark NLRtracker alongside five other tools, and NLRtracker is performing well in terms of

sensitivity and specificity. However, the authors do not explicitly access how well NLRtracker is able to correctly classify the domain architecture, one of the main reasons to develop this novel tool. Providing
this additional benchmark, for which the authors could likely use the predictions in Arabidopsis that overlap with their Arabidopsis-RefPlantNLR annotations, is essential to ascertain the usability of NLRtracker and its performance in contrast to the other established tools.

We thank the reviewer for this suggestion and now make more explicit how the NLRtracker domain architecture functions. We used InterProScan to functionally annotate the RefPlantNLR dataset. We initially manually converted this to the domain architecture annotation, but subsequently made early versions of NLRtracker to automate this process. Hence the domain architecture annotation produced by NLRtracker on the RefPlantNLR dataset is identical to the RefPlantNLR domain architecture. We now state this more clearly in the text. As InterProScan is updated so the domain architecture in RefPlantNLR will be updated. For example, currently there is no InterPro signature for the CCG10 type N-terminal domain and we do not annotate this domain. Recent updates to InterProScan (specifically the update of Gene3D v.4.2.0 to v.4.3.0) have allowed us to improve our domain architecture annotation. The annotation of the Rx-type CC domain is improved because the new version of Gene3D has a signature for this domain, and we extended the extracted NB-ARC domain to include the Winged-Helix domain as the current Gene3D model for this domain is improved.

For the benchmarking of functional annotation, we only considered the canonical plant NLR domains as none of the other tools functionally annotated integrated domains or duplicated domains. The main issue with DRAGO2, RRGPredictor, and RGAugury in the functional annotation of canonical NLR domains seems to be that they use Coils (v2.2.1) to predict CC domains. However, this program does not distinguish between the different types of CC domain. Given the fact that DRAGO2 fails to annotate the CC domain in 145/331 CC-NLRs which get a Rx-type CC domain annotated using InterProScan, and 6/7 CCR-NLRs which get annotated with the RPW8-type CC using InterProScan we conclude that Coils is not the best program for this job.

For the RefPlantNLR dataset we now also include the use of LRRpredictor [1] to annotate individual LRR repeats (supplemental data S10, **NEW**) and the prediction of the C-terminal jelly roll/Ig-like domain (C-JID) [2] (supplemental data S11, **NEW**), and these domains can be visualized on the amino acid sequence with the provided GFF annotation (supplemental dataset 12) or using the refplantnlR R package.

Detailed comments and suggestions:
p4: 'To validate the recovered sequences…' ◊ could the authors please indicate how many sequences were in their initial set?

We now restructured the text to emphasize that we manually crawled through the literature specifically looking for NLRs. We did not encounter any article in which a gene was reported to be an NLR which it did not turn out to be.

p4: 'In addition to the 442 NLRs present…' ◊ How many non-plant sequences or sequences with additional features were later added to the dataset?

We added 5 bacterial, 1 archaeal, and 7 metazoan NB-ARC containing proteins as a separate dataset (supplemental dataset S4). We chose these NLRs as they are functionally characterized and/or had their three-dimensional structure elucidated. We now add the extracted NB-ARC domains from these sequences as a supplemental dataset (dataset S13, **NEW**) and have used these to root the RefPlantNLR phylogeny.

Figure 1: For non-experts, it would be helpful to display a representative protein domain architecture for the four subclades discussed in Figure 1. Furthermore, it would be instructive if the authors would provide higher level taxonomic information for the plant species shown in the phylogenetic trees; for example, highlight monocots and dicots or the different plant clades.

We thank the reviewer for these suggestions. We have now added the representative domain architecture (Figure 1A, **NEW**). Additionally, we more clearly illustrate the NB-ARC domain and highlight where the NLR-motifs align to the three distinct structural features of the NB-ARC domain (P-loop-containing NTPase domain, helical domain of APAF1, and the Winged-Helix domain)

To Figure 1 we now added higher level taxonomic information

p5: 'In total, 31 plant genera representing 11 taxonomic orders are listed…' ◊ It doesn't seem to be surprising that functional characterization of NLRs has been largely focused on a few model and crop species and thus does not represent the plant biodiversity. How does the focus on a small subset of plant biodiversity impact the authors' (and others') approaches to predict and describe NLRs? For example, what can be learnt from the size distributions of NLRs based on this small subset? Could the authors speculate how this limitation could be overcome in the future?

Although we understand the concern of reviewer, the major limitations depends on the interests of the specific researcher. From a crop genetics point-of-view the current understanding may be good enough. From an evolutionary point of view the dataset would be limited and full NLRome predictions are needed.

We hope the reviewer understands our approach which aims at reducing bias in the computational predictions of NLR by first curating the (large) set of experimentally validated NLRs. Otherwise, it's a chicken and egg circular exercise. This is still useful to provide a foundation for future studies and a benchmarking tool for unbiased predictions. This can of course evolve as more experimental data accumulates.

The NLR motifs used here do not work as well on NLRs from non-flowering plants. We await the functional validation of non-flowering plant NLRs before truly delving in their analyses.

p8: 'We selected the 5 most popular…' ◊ How did the authors define 'most popular' in this context? Could the authors add a brief explanation on how the five tools differ and extend the description that is already provided?

We have now changed the wording “5 most popular” to remove most popular. These are the only tools known to the authors for extracting NLRs. We additionally extended the description of these methods used for functional annotation in Table 1

p11: 'In addition to the four main subclades of NLRs, we…' ◊ The authors report an additional TIR-NB-ARC (TN) class and note that this class clusters separately in a phylogenetic analysis. It is unclear if thus phylogenetic analysis in Meyers et al. 2002 or if it is part of the research reported here.

We have now clarified that Meyers et al. noticed the different gene structure, but that the clustering is part of the research reported here. These proteins get annotated with the PF00931 NB-ARC signature (0/4 Arabidopsis, 2/3 tomato, and 2/4 rice). In a phylogenetic analysis including the non-plant outgroup these NLRs cluster with the non-plant NLRs. We now include this phylogeny (Figure S4, **NEW**). They meet our criteria of NLRs, having an NB-ARC domain and one additional domain, but they lack an identifiable super-structure forming repeat. Finally, structural searches against PDB using the recently released AlphaFold2 predictions identify APAF1, and not plant NLRs, as the first hit.

This TN class of NLRs appears conserved in all plant genomes we looked at, including *Selaginella moellendorffii* (a lycophyte), *Physcomitrium* (mosses) and *Marchantia* (liverworts), similar to the recently described plant MLKL family [3]. Whether this subgroup of NLRs has a role in immunity is not currently established. Please note that they may have other functions.

p12: How do the authors define 'genuine NLR' in the context of their benchmarking? Related, to determine specificity, one needs to obtain false positive calls but how these are defined based on the genuine NLRs is not clear. For example, the authors mention 'These false positives were predominantly proteins containing a P-loop containing nucleoside triphosphate hydrolase domain unrelated to the NB-ARC domain.' How did the authors determine that the P-loop domain was unrelated to the NB-ARC domain?

The NB-ARC domain belongs to the P-loop containing NTPase superfamily. One major subclass is called the STAND (signal transduction ATPases with numerous domains) class. This class encompasses the NB-ARC domain, as well as the NACHT domain, but also adenylyl cyclases. In the case of these false positives the P-loop containing NTPase signature overlapped with other signatures belonging to specific subcategories of the STAND class. We now clearly state which STAND class subcategory these false positives belong to.

Table 1: The authors should add the respective references to each tool to the table

We have added these references.

NLRTracker: The developed pipeline relies on identification of known sequence motifs or profiles (i.e., PFAM domains) in the predicted proteomes. This process typically involves setting cutoffs to distinguish true positive from false positive matches, and thus influence the number of identified NLRs and quality of these predictions. The authors

need to define which cutoffs they applied (for instance in InterproScan) and if identical cutoffs were applied for each domain or if domain specific cutoffs that reflect diversity within a domain have been used. This might also be related to potential false positives discussed above. Similarly, do the authors apply any length related cutoffs, e.g., in Fig 3C some NLRs have very small NB-ARC domains, to retrieve and classify sequences into NLRs.

For NLRtracker we use the default output of InterProScan, which already applies a post-processing filtering step. For the MEME search using the predefined NLR motifs we now specify the cutoff in the M&M:

"We did not apply additional cut-offs to the InterProScan output. For the MEME output we filtered for hits with a score ≥ 60.0 and a qvalue ≤ 0.01. Additionally, for NLR extraction using the linker and MHD motif we applied a more stringent cut-off requiring a score ≥ 85.0."

We have not applied any other criteria as we try to maximize the output of *potentially* functional NLRs. This output should be manually inspected. Additionally, this output can be used to assess whether the annotation of a genome correctly annotates NLRs: in case of many seemingly truncated NLRs or fused NLRs it is fair to assume that the genome annotation requires additional curation for this family of genes.

Reviewer #2:
[identifies himself as Bingyu Zhao]

In this manuscript, the authors described a plant NLR database (RefPlantNLR) with 442 NLRs that have been experimentally validated. Five NLR-annotation tools were benchmarked by using the RefPlantNLR database. DRAGO2 is the most sensitive tool for the identification of NLRs. However, its annotation specificity is low. The other tools also have pros and cons. Therefore, the authors decide to develop a new pipeline, NLRtracker, for extraction and annotation of plant NLRs. Comparing to other tools, NLRtracker has significantly improved both sensitivity and specificity for extraction and annotation of plant NLRs. The authors also provide all curated datasets and the scripts used to analyze the dataset.
The RefPlantNLR database and the NLRtracker will be a valuable resource for the plant immunity research community, and it is likely to be heavily cited in the future!
The whole experiment was well designed; the data was analyzed with appropriate bioinformatics tools and logically interpreted. The manuscript was very well prepared. I feel it is ready to be accepted for publication!

We thank the reviewer for the kind words.

Two minor suggestions:
Fig3c, it looks like there were 3 kinds of NB-ARC domains. Please add the information in the figure legend. If they are referring to the description on page 15, the authors can add a few sentences to refer to figure 3c.

We had coloured the different NB-ARC domains based on the phylogenetic subclade (CC-NLR, TIR-NLR, etc.). We have now removed this colouring to avoid confusion.

Page 8, following "that NLR-Annotator, delete an extra space

Done.

Reviewer #3:
[identifies himself as Detlef Weigel]

PLoS Biology PBIOLOGY-D-21-00318_R1

I apologize for the time it has taken me to review this work, but things are currently unpredictable.

The current work makes a very solid contribution to the exciting field of (plant) NLR biology. The authors have been extremely careful to compile an excellent set of NLR sequences from genes that have been shown to have some sort of function (overwhelmingly, conferring disease resistance) in different plant species. The collection is currently biased towards A. thaliana, but it is a living collection of sequences and I have full confidence that the authors will continuously update it, and that this bias will soon disappear, as positional cloning is quickly becoming routine even in difficult crop species.

We thank the reviewer for the kind words and would like to highlight that as mentioned above we indeed aim to provide periodic updates to the RefPlantNLR dataset through Zenodo. This will ensure reproducibility and availability of the underlying data. Indeed, in between the last and current version we have added 38 additional entries.

My major concern is that the value of the resource is limited because it seems to consist primarily of downloadable flat files, instead of an interactive database that can be used to explore domain structures and sequence similarities. I would strongly urge the authors to build such a resource.

To address this concern we have now generated an R package called refplantnlR to visualize the domain architecture of the RefPlantNLR entries (see GitHub for explanation). Additionally, this package takes input from NLRtracker to visualize the NLRtracker output and it comes pre-packaged with the precomputed NLRtracker output on all plant NCBI RefSeq proteomes.

Although we agree that an interactive database may open this resource to a larger audience, we feel that this is outside of the scope of this paper. Currently we are focusing on reproducible science and providing a dataset which can be cited and used by others. Providing updates through Zenodo will put a DOI on the different versions and link it to the published article which allows for reproducible science. We believe that these flat files should provide an accessible format for most biologists that want to use this resource. An interactive database is likely to result in users downloading the data from there without version tracking or citation, in addition to incomplete datasets being used. We have consulted with our resident bioinformatics expert (Dan Maclean) and the estimated costs of building such a resource, responding to comments by users,

and maintaining it for 10 years are beyond the financial commitment we can allocate to this project. Additionally, the hours involved in maintaining such an interactive database are prohibitive.

To perhaps address the concern of the reviewer about the value of the dataset, we plan to post tutorial videos on how best to use the flat files, for instance through tools like Geneious and other applications such as how to add new NLRs to the existing phylogenies. We hope that such tutorial, which will also be referenced through the Zenodo OpenPlantNLR community will broaden the user base of RefPlantNLR.

There is not much to criticize regarding the presented data themselves, as the analyses are straightforward (even if they involved a very considerable amount of work). However, my opinion is that more could be done with the dataset without too much extra effort, and that such additional analyses would make the study considerably more appealing.

1. An important question in plant NLRology is how many of the NLRs have a bona fide function, and how many are a just byproduct of rampant sequence diversification. The authors can now ask whether the RefPlantNLR set is a random subset of annotated NLRs in the respective species, at least for the four top species (Arabidopsis, tomato, rice, wheat), or whether the RefPlantNLR set has properties that sets them apart.

We now include for Arabidopsis, tomato, and rice a graph showing the distribution of the main subclades of NLRs in RefPlantNLR as compared to the NLRome. For Arabidopsis we only used the characterized NLRs from Col-0, while for the tomato and rice NLRs we identified the likely homologs for the RefPlantNLR entries in the reference genomes by phylogeny. We also compared the number of NLRs in these species with an integrated domain. It seems that NLRs with integrated domains are slightly overrepresented in RefPlantNLR. Whether this reflects the fact that these are more often involved disease resistance, or whether there is a degree of research bias is not certain. Finally, for Arabidopsis, whose genes are manually curated we compare the NLR domain architecture in the RefPlantNLR dataset vs the NLRs from the reference genome.

For wheat we extract 2749 potential NLRs using NLRtracker from the annotated high-confidence geneset (iwgsc v2.1 HC annotation) and we have omitted it from this analysis.

2. Another related question is the population frequency of NLR alleles with likely identical function. For Arabidopsis, a collection of NLR genes and alleles from dozens of strains has been published, and the authors can now ask both whether the distribution of orthogroups defined by RefPlantNLR members across these strains is different (or not) from random NLRs, and whether the sequence variation within the RefPlantNLR orthogroups is significantly different from NLRs without known function. Perhaps one can use data from the recent Prighozin and Krasileva paper for this purpose.

This sounds like a perfect use of the RefPlantNLR dataset by the community. These analyses would be great in the context of a paper which focuses on Arabidopsis or inter-species-specific differences.

One message we hope to convey with this paper is the need to explore NLR diversity outside of a small set of model organisms and crops. We feel that the proposed analysis would detract from this message and don't see where it would currently fit in the manuscript.

I have two further suggestions/criticisms. The first one is whether genes/alleles that are only defined by autoimmunity including hybrid necrosis should be included as RefPlantNLRs. So far, at least for genes with induced autoimmune alleles, we only know that they can be mutated in a way that they become spontaneously active - but wouldn't this likely apply also to many other NLRs? I admit, there might be something special about these genes, because these, and not other genes, showed up in mutant screens.

We considered two classes of autoimmune mutations: 1) mutations in the NLR gene itself, or 2) mutations in genes required for NLR function. For 1) this could be for example DV mutations in the MHD motif, or mutations such as in slh1. While these mutations show that the NLR is signalling capable it does not indicate whether it has a ligand which can activate the non-mutant NLR in a physiologically relevant situation. SLH1 is required for resistance towards pathogens translocating AvrRps4, PopP2, and other as-of-yet uncharacterized effectors. For 2) an example would be KO of RIN4 which leads to RPS2 activation. This shows that RIN4 is somehow involved in RPS2 activation (which it is) but does not necessarily prove that RPS2 has a physiologically relevant function (which in this case it does). In the case of hybrid necrosis it could also be 3) inadvertent interaction of components which normally do not coexist which do not shed light on the physiological function of these NLRs.

Currently we have decided to include all these examples as at least from a biochemical perspective these NLRs are signalling capable, and they might have a physiological function in disease resistance. In the supplemental table we highlight which pathogens are recognized by an NLR, or whether they have only been characterized in autoimmunity. Depending on the specific case users can decide to filter these out or keep them in.

A more important criticism concerns the NLR annotation tools. All of these require independent annotation of gene models to derive the final NLR genes, regardless of whether they use CDS or genomic sequences as inputs. Deriving correct gene models for NLR genes is difficult, and often requires considerable manual curation. This should at least be clearly discussed, including perhaps how NLR annotation tools including the new one introduced here can potentially be used to address these difficulties.

We agree that NLR gene-model annotation is currently not straight-forward. NLRtracker provides functional annotation for these genes, and as such this information can be used to quickly assess whether a given NLR gene model is potentially split (in case it lacks certain key features) or whether it is fused. This is especially powerful when the output of NLRtracker (fasta files of NLRs and gff3 of functional annotation) is combined with a tool to visualize the annotation on the sequence, such as the refplantnlR R package. Additionally, NLRtracker is currently the only tool that provides functional annotation for integrated domains, and it classifies the most commonly identified integrated domains. In Figure S7 it can be seen that many of these integrated

domains are potential transposon-related domains. While some of these may be genuine integrations-or gene disruptions-the majority likely reflects miss-annotations and fusions to flanking transposon sequences. We now discuss the use of NLRtracker to assess whether a given gene model is correct in the text.

Reviewer #4:

I come in at the first revision stage as a new reviewer. This paper is of interest to the community of plant pathology and likely more broadly to plant biology. I do not think it has major appeal outside these areas. Similar work e.g. NLRannotator http://www.plantphysiol.org/content/183/2/468 have been published elsewhere.

Here are my concerns and questions (it's a shame that line numbers are missing):

* NLRtracker works on what level? Identified loci? CDS and protein? This is not clear on the github page and in the abstract. The github page also lacks a reuse license. Is it an extractor or annotator? I see the figure 6 shows it works on transcripts/AA sequences. I think this should be clarified up front. I think the field would really benefit from a pipeline that extracts loci from raw genomic sequence, annotates gene models on these with a focus on NLRs, and functionally annotates the resulting protein sequences as NLRtracker does. This would be important to standardize the whole annotation pipeline as the diversity analysis between papers and species falls already flat if not all NLR gene models are pulled out in the first place. This is not a required for the authors to design this pipeline.

We have now clarified on the Github page that NLRtracker works on transcript/AA level and added this in the abstract. We have added the MIT license for redistribution. NLRtracker extracts the NLRs from transcript/AA sequences and functionally annotates these with protein domains. We agree that an entire pipeline from genomic sequence to functionally annotated proteins would be great to have some day.

* The author should be more careful in what context they use annotation e.g. genome annotation with genes or functional annotation of proteins. This will make reading the manuscript easier. Later on the author use the term NLR-retrieval. Consistent usage of terms throughout the text would be great and really help the flow.

We have now removed NLR-retrieval from the text and consistently used functional annotation.

* Paragraph:
"These various tools use pre-defined motifs to
classify sequences as NLRs, but they differ in the methods and pipelines. NLR-Annotator -an
extension of NLR-Parser-and NLGenomeSweeper, can also use unannotated genome sequences as input to predict the genomic locations of NLRs (Steuernagel et al., 2020; Toda et
al., 2020). This output then requires manual annotation to extract the final gene-models and

some of the annotated loci may represent partial or pseudogenized genes. "

It is not correct that one has to manually annotate these loci. One can run gene prediction tools on extended identified loci such as braker etc.

We disagree that one can simply and easily run gene prediction tools to correctly extract NLR gene models without any form of manual curation afterwards, although the output from these tools does provide a good starting point. Even in the presence of a well-annotated reference genome, targeted long-read sequencing of NLR transcripts (SMRT RenSeq), RNAseq under pathogen-stress conditions (as many of these genes are inducibly transcribed and spliced), and gene prediction tools which are well-calibrated for a specific organism NLR annotation still requires manual validation [4]. Specifically, with NLRs the most common types of miss-annotation are fused transcripts (due to the tendency to occur in tandem repeats and near transposable elements), split transcripts, and missing transcripts. Additionally, miss-annotated splicing in tandemly repeated regions could result in chimeric transcript annotation.

* The title paragraph headers could be more descriptive.

We have now added more descriptive paragraph headers.

* It would be nice to have a table in the text that clearly provides all domains (in whatever combination) are required to be found in a protein to call it a NLR. It is a bit confusing from the text. I see it is added to the methods section somewhat and it could be clearer.

We have now included this in Figure 1A.

* Figure 3 a and b: What do all the letter codes below the graph mean?

The letter codes are described in the figure legend and are abbreviations of the various domains found in NLRs. We now moved the legend up, so it is clearer that the legend is for the entire figure and highlighted the letter codes in bold.

* It would be worthwhile to compare the methods of functional annotation for all the annotation tools bench marked in this manuscript. Also this work does only benchmark falls negative and not false positive rates. This might be useful to know as well.

We now highlight in Table 1 the methods used by the various tools for functional annotation and mention this in the text.

As we note RefPlantNLR cannot be used for benchmarking false positives. It is for this reason that we included the Arabidopsis, tomato, and rice proteomes to also look for false positive rates which we report in Figure S5.

* How is domain prediction accuracy defined? This is unclear. Overall the whole benchmarking section is a bit confusing as not all the tools do the same e.g. NLR-annotator does only loci and rough motifs but does not identify gene models on these loci. How does this compare in the annotation specificity with others which work on

protein sequences? Also it is unclear why it is split in two section with one with and one without NLRtracker. The whole benchmarking section will benefit from a restructure for clarity.

The benchmarking is in two sections because of benchmarking on the RefPlantNLR dataset first (sensitivity) and next on the Arabidopsis, tomato, and rice proteomes (sensitivity & accuracy) (see Figure S5 for the false-positives). NLRtracker was developed using RefPlantNLR and the domain annotations which were found to be informative (described in the Material & Methods). Running NLRtracker on RefPlantNLR gives the exact same output as the current annotation of RefPlantNLR. We now highlight this more clearly in the text.

Both NLR-Annotator and NLGenomeSweeper work only on nucleotide sequences. We ran these two tools both on the CDS and the extracted genomic loci (where available), and in the direct comparisons with other tools we only compare the RefPlantNLR entries for which we had an associated CDS entry. We highlight this more clearly in the text. We reasoned that CDS and AA sequences should be equivalent, and that NLR-Annotator should correctly extract all NLRs from a CDS file. While NLR-Annotator performs similarly on both CDS/genomic loci NLGenomeSweeper performs considerably worse on genomic loci, both for functional annotation and for NLR extraction. NLR-Annotator fails to extract some NLRs, notably the CCR-NLRs. It also extracts NLRs with duplicated NB-ARC domains twice, which is expected based on the design of NLR-Annotator.

While NLR-Annotator does not strictly output functional annotation, nor was it meant to, the MEME motifs overlap with specific protein domains (specifically, Rx-type CC, TIR, NB-ARC, and LRR), and we made a small R script to convert the motif output to functional annotation (included in Appendix S1). Surprisingly, converting these motifs to functional annotation outperforms the other tools in accuracy-except for NLRtracker which would have a 100% accuracy on the RefPlantNLR dataset and which is also capable of functionally annotating integrated domains and the Late blight R1 domain, as well as duplicated NB-ARC domains, and distinguish between the different type of CC domains.

Overall the work seems well done (while wordy and difficult to follow at times) and contributes to the advancement of the field.

1. Martin EC, Sukarta OCA, Spiridon L, Grigore LG, Constantinescu V, Tacutu R, et al. LRRpredictor—a new LRR motif detection method for irregular motifs of plant NLR proteins using an ensemble of classifiers. Genes. 2020;11: 286. doi:10.3390/genes11030286

2. Ma S, Lapin D, Liu L, Sun Y, Song W, Zhang X, et al. Direct pathogen-induced assembly of an NLR immune receptor complex to form a holoenzyme. Science. 2020;370. doi:10.1126/science.abe3069

3. Mahdi LK, Huang M, Zhang X, Nakano RT, Kopp LB, Saur IML, et al. Discovery of a family of mixed lineage kinase domain-like proteins in plants and their role in innate immune signaling. Cell Host Microbe. 2020 [cited 4 Dec 2020]. doi:10.1016/j.chom.2020.08.012

4. van de Weyer A-L, Monteiro F, Furzer OJ, Nishimura MT, Cevik V, Witek K, et al. A species-wide inventory of NLR genes and alleles in *Arabidopsis thaliana*. Cell. 2019;178: 1260-1272.e14. doi:10.1016/j.cell.2019.07.038
