# Supplementary material for: RefPlantNLR is a comprehensive collection of experimentally validated plant disease resistance proteins from the NLR family
Source: PLoS Biol. 2021 Oct 20;19(10):e3001124. doi: 10.1371/journal.pbio.3001124 (PMC8559963; doi:10.1371/journal.pbio.3001124)
Supplement: Attachment — Submitted filename: 20210909_Response.docx [file pbio.3001124.s038.docx]

Dear Sophien,
 
Thank you for submitting your revised Methods and Resources entitled "RefPlantNLR: a comprehensive collection of experimentally validated plant NLRs" for publication in PLOS Biology. I have now obtained advice from two of the original reviewers and have discussed their comments with the Academic Editor. 

Based on the reviews (attached below), we will probably accept this manuscript for publication, provided you satisfactorily address the remaining point raised by Reviewer 1. Please also make sure to address the policy-related requests stated below.

We have addressed the reviewers comments and increased the font size and added the species to the phylogeny figure. Additionally we have added the PDF as a new supplementary dataset as it might be easier to search and find specific sequences in this way.

We have also addressed the policy requests.

In addition, we would like you to consider a suggestion to improve the title:

"RefPlantNLR is a comprehensive collection of experimentally validated plant disease resistance proteins from the NLR family"

We have changed the title

As you address these items, please take this last chance to review your reference list to ensure that it is complete and correct. If you have cited papers that have been retracted, please include the rationale for doing so in the manuscript text, or remove these references and replace them with relevant current references. Any changes to the reference list should be mentioned in the cover letter that accompanies your revised manuscript.

We expect to receive your revised manuscript within two weeks. 

To submit your revision, please go to <https://www.editorialmanager.com/pbiology/> and log in as an Author. Click the link labelled 'Submissions Needing Revision' to find your submission record. Your revised submission must include the following:

-  a cover letter that should detail your responses to any editorial requests, if applicable, and whether changes have been made to the reference list


NOTE: If Supporting Information files are included with your article, note that these are not copyedited and will be published as they are submitted. Please ensure that these files are legible and of high quality (at least 300 dpi) in an easily accessible file format. For this reason, please be aware that any references listed in an SI file will not be indexed. For more information, see our Supporting Information guidelines:
<https://journals.plos.org/plosbiology/s/supporting-information>  

*Published Peer Review History*
Please note that you may have the opportunity to make the peer review history publicly available. The record will include editor decision letters (with reviews) and your responses to reviewer comments. If eligible, we will contact you to opt in or out. Please see here for more details:
<https://blogs.plos.org/plos/2019/05/plos-journals-now-open-for-published-peer-review/>

*Early Version*
Please note that an uncorrected proof of your manuscript will be published online ahead of the final version, unless you opted out when submitting your manuscript. If, for any reason, you do not want an earlier version of your manuscript published online, uncheck the box. Should you, your institution's press office or the journal office choose to press release your paper, you will automatically be opted out of early publication. We ask that you notify us as soon as possible if you or your institution is planning to press release the article.

*Protocols deposition*
To enhance the reproducibility of your results, we recommend that if applicable you deposit your laboratory protocols in protocols.io, where a protocol can be assigned its own identifier (DOI) such that it can be cited independently in the future. Additionally, PLOS ONE offers an option for publishing peer-reviewed Lab Protocol articles, which describe protocols hosted on protocols.io. Read more information on sharing protocols at <https://plos.org/protocols?utm_medium=editorial-email&utm_source=authorletters&utm_campaign=protocols>


Please do not hesitate to contact me should you have any questions.

Best wishes,

Ines
--
Ines Alvarez-Garcia, PhD,
Senior Editor,
[ialvarez-garcia@plos.org](mailto:ialvarez-garcia@plos.org),
PLOS Biology

------------------------------------------------------------------------

DATA POLICY: IMPORTANT - PLEASE READ

You may be aware of the PLOS Data Policy, which requires that all data be made available without restriction: <http://journals.plos.org/plosbiology/s/data-availability>. For more information, please also see this editorial: <http://dx.doi.org/10.1371/journal.pbio.1001797> 

Note that we do not require all raw data. Rather, we ask that all individual quantitative observations that underlie the data summarized in the figures and results of your paper be made available in one of the following forms:

1) Supplementary files (e.g., excel). Please ensure that all data files are uploaded as 'Supporting Information' and are invariably referred to (in the manuscript, figure legends, and the Description field when uploading your files) using the following format verbatim: S1 Data, S2 Data, etc. Multiple panels of a single or even several figures can be included as multiple sheets in one excel file that is saved using exactly the following convention: S1_Data.xlsx (using an underscore).

2) Deposition in a publicly available repository. Please also provide the accession code or a reviewer link so that we may view your data before publication. 

Regardless of the method selected, please ensure that you provide the individual numerical values that underlie the summary data displayed in the following figure panels as they are essential for readers to assess your analysis and to reproduce it:

Fig. 1B-E; Fig. 2A-D; Fig. 3A, B; Fig. 5A; Fig. S1A-C; Fig S4; Fig. S6A, B and Fig. S7

NOTE: the numerical data provided should include all replicates AND the way in which the plotted mean and errors were derived (it should not present only the mean/average values).

Please also ensure that figure legends in your manuscript include information on WHERE THE UNDERLYING DATA CAN BE FOUND, and ensure your supplemental data file/s has a legend.

Please ensure that your Data Statement in the submission system accurately describes where your data can be found.


------------------------------------------------------------------------

Reviewers' comments:

Rev. 1:
The authors addressed my main concerns and comments raised by the initial submission. I am particularly happy that the authors packaged some of the visualisation capacities into a R package, which has been made accessible to the community via github. I can follow the authors' arguments to provide the raw data as flat files rather than an interactive database given the cost and efforts associated with the development and maintenance of such as resource. Thus, I have no further comments/concerns or concerns related to the analyses. However, I would like to encourage the authors to adjust the font size in the phylogenetic tree (Figure 4) to ensure readability of sequence and motif names. Furthermore, it might be helpful to highlight the species for each sequence in the phylogenetic tree.


Rev. 3:
I thank the authors for diligently responding to my suggestions. This will be a landmark publication for the plant immunity field.

__________________________________________________
In compliance with data protection regulations, you may request that we remove your personal registration details at any time.  (Use the following URL: <https://www.editorialmanager.com/pbiology/login.asp?a=r>). Please contact the publication office if you have any questions.
